# Cancer burden in China: trends, risk factors and prevention

**DOI:** 10.20892/j.issn.2095-3941.2020.0387

**Published:** 2020-12-15

**Authors:** Dianqin Sun, He Li, Maomao Cao, Siyi He, Lin Lei, Ji Peng, Wanqing Chen

**Affiliations:** 1 Department of Cancer Screening, National Cancer Center/National Clinical Research Center for Cancer/Cancer Hospital, Chinese Academy of Medical Sciences and Peking Union Medical College, Beijing 100021, China; 2 Department of Cancer Prevention and Control, Shenzhen Center for Chronic Disease Control, Shenzhen 518020, China

**Keywords:** Cancer burden, time trends, risk factor, prevention, China

## Abstract

As the most populous country in the world, China has made strides in health promotion in the past few decades. With the aging population, the burden of cancer in China continues to grow. Changes in risk factors for cancer, especially diet, obesity, diabetes, and air pollution, continue to fuel the shift of cancer transition in China. The burden of upper gastrointestinal cancer in China is decreasing, but still heavy. The rising burden of colorectal, prostate, and breast cancers is also significant. Lung cancer became the top cause of cancer-related deaths, together with smoking as the most important contributor to cancer deaths. The Chinese government has taken several approaches to control cancer and cancer-related risk factors. Many achievements have been made, but some challenges remain. Health China 2030 is ambitious and depicts a bright vision of the future for cancer control in China. The decrease in the cancer burden in China will require cross-sector collaboration and coordinated efforts on primary and secondary preventions by governments, public health organizations, and individuals. In this review, we describe the trends of cancer burden and discuss cancer-related risk factors in China, identifying strategies to reduce the burden of cancer in China.

## Introduction

With the largest population in the world, China has made extensive progress in promoting health since the 1950s. The changes in lifestyles, age structure, and living conditions in the Chinese population have led to a shift in disease burden from infectious diseases to non-communicable diseases (NCDs). Cancer has now become the leading cause of death in China^1^. According to the National Central Cancer Registry of China (NCCR), approximately 2.338 million cancer deaths occurred in 2015^2^. Furthermore, the burden of cancer is expected to continue to increase.

Since Percivall Pott identified the cause of “the chimney- sweepers’ cancer,” attributing cancer to specific causes has become the basis of cancer prevention^3^. In 1981, Doll and Peto^4^ published their seminal work describing the avoidable causes of cancer. Epidemiological studies have currently identified numerous behavioral, clinical, and environmental risk factors for cancer. Dramatic changes in the prevalence of these risk factors have been observed in the past 30 years, which led to the ongoing cancer transition in China, including a reduction in upper gastrointestinal cancer and increases in westernized lifestyle-related colorectal, prostate, and breast cancers^5^. The changes that are needed to curb the cancer epidemic require sustained interventions for primary prevention targeting of risk factors.

This review aimed to provide an overview of cancer burden trends, cancer-associated risk factors, and prevention strategies in China. Although many potential risk factors for cancer exist, we discussed well-established risk factors. We reviewed the current situation and changes in these risk factors over the past 3 decades. We also reviewed key policies on cancer control in China, and discuss strategies for nationwide cancer prevention.

## Time trends of the cancer burden

In this part, we reviewed data from the second and the third nationwide retrospective surveys of mortality^6,7^, and cancer registry reports by the NCCR^2,8^. We also searched the online cancer database of the Global Burden of Disease study 2017 (GBD 2017)^9^. The GBD study was initiated by the Institute for Health Metrics and Evaluation, which provides age-sex-specific disability-adjusted life years (DALYs) for countries across the world. The age-standardized DALY rates were based on the GBD reference population^10^. Cancer survival data^11^ from the NCCR were retrieved to provide a complete picture of the time trends of the cancer burden in China. Age-standardized rates were calculated using the International Cancer Survival Standards^12^.

### Incidence, mortality, and DALYs

Data from 22 Chinese registries showed that the age-standardized cancer incidence rate (using the world standard population^13^) increased from 2000 to 2011, with a significant rise in females and a stable trend among males^8^. Significantly decreased trends in the age-standardized incidence rates of liver, stomach, and esophageal cancers were observed for both sexes. This trend resulted from widespread hepatitis B virus vaccination^14^, reduced aflatoxin exposure^15^, better food preservation practices, and better living conditions^16^. The age-standardized incidence rates of colorectal cancer experienced a significant upward trend for both sexes. For males, the age-standardized incidence rate of prostate cancer increased, with an annual percentage change of 12.6% from 2000 to 2011. The age-standardized incidence rates of breast, cervix, and ovary cancers also rose sharply.

During the past 20 years, the crude cancer mortality rate increased from 108.3 per 100,000 in 1990–1992 to 170.1 per 100,000 in 2015, while the age-standardized mortality rate (using the 1982 Chinese national census population) decreased from 94.4 per 100,000 to 77.9 per 100,000^5^. These opposite changes suggested that the rise of cancer mortality was mainly attributable to the aging population. **Table 1** shows the changing mortality rates of the 10 most common cancer types in China between 1990 and 2015. The past decades have seen a significant decline in either crude or age-standardized mortality rates of stomach, esophageal, and liver cancers. The rising mortality rates of colorectal cancer, breast cancer, and pancreatic cancer also suggested a transition in cancer types.

**Table 1:** Mortality rates of the 10 most common cancer types in China from 1990 to 2015

Rank by death cases	Year 1990–1992^6^			Year 2004–2005^7^			Year 2015^2^		
	Site	Crude rate (per 100,000)	Standardized rate* (per 100,000)	Site	Crude rate (per 100,000)	Standardized rate* (per 100,000)	Site	Crude rate (per 100,000)	Standardized rate* (per 100,000)
1	Stomach	25.2	21.8	Lung	30.8	20.2	Lung	45.9	20.2
2	Liver	20.4	17.8	Liver	26.3	17.9	Liver	23.7	11.4
3	Lung	17.5	15.2	Stomach	24.7	16.2	Stomach	21.2	9.4
4	Esophagus	17.4	15.0	Esophagus	15.2	10.0	Esophagus	13.7	6.0
5	Colorectum	5.3	4.5	Colorectum	7.3	4.7	Colorectum	13.6	5.8
6	Leukemia	3.6	3.5	Leukemia	3.8	3.4	Pancreas	6.2	2.6
7	Cervix	3.9	3.3	Brain and nervous system	3.1	2.4	Female breast	10.5	5.0
8	Nasopharynx	1.7	1.5	Female breast	5.9	4.0	Brain and nervous system	4.1	2.4
9	Female breast	3.5	3.0	Pancreas	2.6	1.7	Leukemia	4.0	2.6
10	Bladder	1.0	0.9	Bone	1.7	1.2	Lymphoma	3.6	1.3

*Age-standardized rates were calculated using the 1982 Chinese national census population.

**Figure 1:**
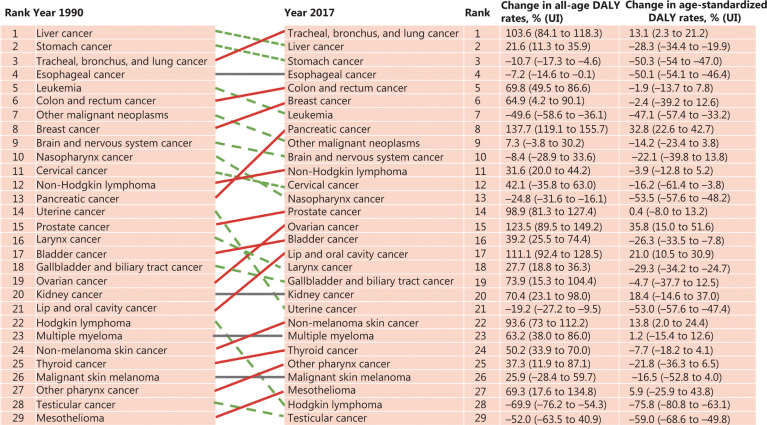
Rank changes in disability-adjusted life years (DALYs) attributable to cancers and percentage changes in all-age and age-standardized DALY rates in China from 1990 to 2017. The estimates used to generate this figure were from the Institute for Health Metrics and Evaluation website^9^. Age-standardized rates were calculated using the GBD reference population^10^.

**Figure 1** shows the rank changes in DALYs attributable to cancer, and percentage changes in all-age and age-standardized DALY rates between 1990 and 2017. According to GBD 2017, lung cancer now tops the list, having displaced liver cancer to second place and stomach cancer to third place. The age-standardized DALY rates of stomach cancer and esophageal cancer have decreased by more than half over the last 27 years, but stomach cancer and esophageal cancer are still in the top 4, with very large DALYs. The all-age DALY rates of colorectal cancer and breast cancer have increased by 69.8% and 64.9% since 1990, respectively, leading to an increase in their rankings, to become the fifth and sixth positions, respectively. Pancreatic cancer moved from outside the top 10 to 8th place with a 137.7% increase in all-age DALY rates over this period. The age-standardized DALY rate of uterine cancer decreased by 53%, leading to uterine cancer outside the top 20 in 2017.

### Cancer survival

**Figure 2:**
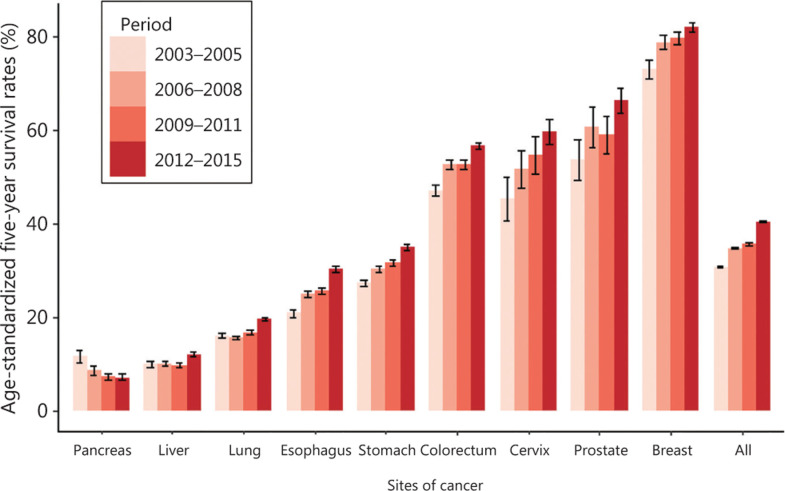
The age-standardized 5-year relative survival rates of common types of cancers in China from 2003 to 2015. The data were from Zeng et al.^11^. Age-standardized rates were calculated using the International Cancer Survival Standards^12^.

**Figure 2** shows the age-standardized 5-year relative cancer survival rates in China from 2003 to 2015^11^. For all combined cancers, the age-standardized 5-year relative survival increased from 30.9% to 40.5% between 2003 and 2015. The 5-year survival rates of common types of cancers generally increased, except for pancreatic cancer. Significant upward trends in the relative survival rates were observed for larynx, esophagus, bone, stomach, corpus uteri, cervix, thyroid, and bladder cancers. The largest improvements in the 5-year relative survival rates were observed in cervix, uterus, thyroid, and bone cancers. The improvement in cancer survival could be attributed to many factors, including improved treatments, increased screening uptake rates, and the expansion of health insurance. In addition, the disparity of urban and countryside rates has been reduced over time, though the gap remains significant. The 5-year relative cancer survival adjusted by age, sex, and cancer type has increased from 34.8% in 2003–2005 to 38.1% in 2012–2015 in urban areas. During the same period, the rates in rural areas increased from 24.6% to 34.5%^11^. Despite these improvements, there is still room for growth in cancer survival in China. Compared with its East Asian neighbors, Japan and the Republic of Korea, China has relatively lower survival rates of breast, prostate, thyroid, colorectal, and stomach cancers. For example, the 5-year survival rate of stomach cancer was less than 36% in China, which has reached approximately 70% in the Republic of Korea^17^. Higher screening uptake rates in Japan and the Republic of Korea are thought to be the main reasons, which involves the inflation of cancer incidence and promotion of the detection of cancer in its early stages^18^.

## Cancer risk factor burden

**Figure 3:**
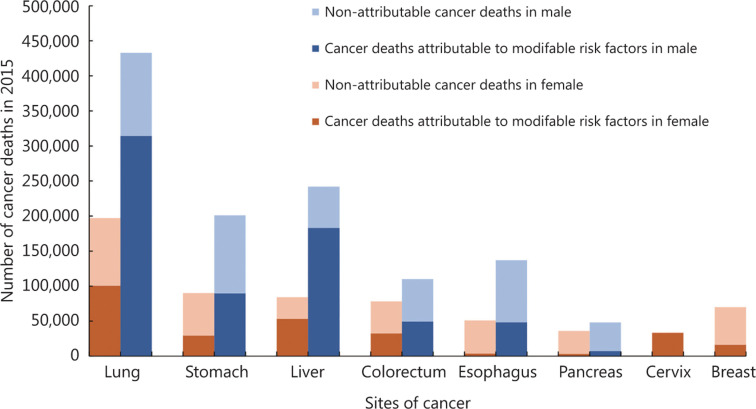
The proportion of cancer deaths that could be attributed to modifiable risk factors in common types of cancer deaths among males and females in China. The data used to generate this figure were from Chen et al.^19^

**Figure 4:**
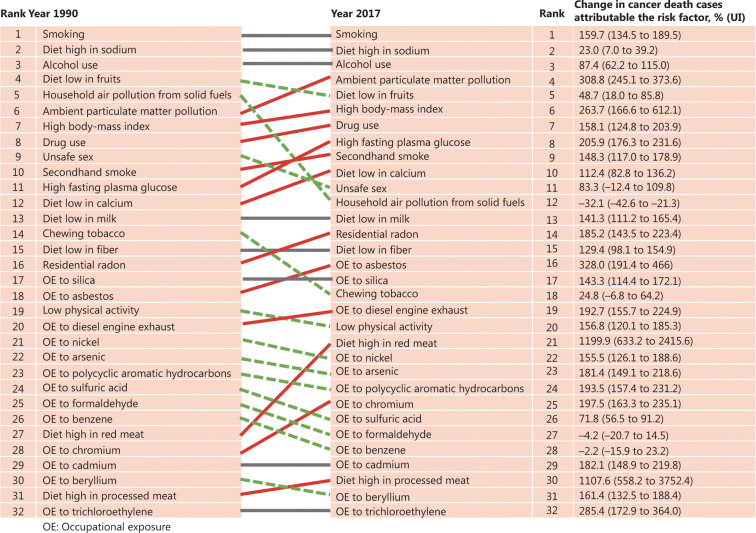
Rank changes in cancer deaths attributable to 32 modifiable risk factors, and percentage changes in cancer deaths attributable to these risk factors in China from 1990 to 2017. The estimates used to generate this figure were from the Institute for Health Metrics and Evaluation website^9^.

In this section, we focused on the established risk factors involving data that are available and robust. To depict the current situation of cancer burden caused by various risk factors in China, we cited data from the National Cancer Center^19,20^. As measured by population attributable fraction, 45% of cancer deaths in China were estimated to be attributed to modifiable risk factors^19^. **Figure 3** presents the proportion of cancer deaths that could be attributed to modifiable risk factors in common types of cancer deaths among males and females. Because of the lack of official data about the impact of time trends of risk factors on the cancer burden in China, we extracted data from the GBD 2017 using the online results tool^9^. **Figure 4** shows the rank changes in risk factors contributing to cancer deaths, and the percentage changes of cancer deaths attributable to risk factors from 1990 to 2017. Detailed methods for the GBD study have been described in previous studies^21^.

### Behavioral risk factors

#### Smoking

Cancer-causing substances in tobacco smoke catalyze the formation of DNA adducts, subsequently resulting in the accumulation of somatic mutations^22^. Since tobacco was established as a carcinogen linked to lung cancer in the 1950s, there has been a large body of evidence showing that tobacco use increases the risk of more than 15 types of cancer. The International Agency for Research on Cancer (IARC) also concludes that passive smoke is carcinogenic^23^. China has the largest number of smokers and consumes over 40% of the world’s tobacco^24^. In China, smoking was estimated to cause 23.8% of cancer deaths among males and 4.8% among females in 2014. Among these deaths, 10.2% of lung cancer deaths in females and 1.4% in males were attributed to passive smoke^20^. Corresponding to substantial geographical variations on smoking prevalence, the smoking-attributable cancer burden ranged from 15% to 27% among males and from 2% to 15% among females in Chinese provinces^20^.

No national picture of smoking prevalence was available in China before the National Survey on Smoking in 1984^25^. Since then, several nationally representative surveys related to tobacco use have been conducted^26–33^. **Table 2** summarizes the smoking prevalence from those surveys, and the results are different even in the same year due to various sampling populations, data collection methods, and study qualities. Although a decline in smoking prevalence during the past 30 years was observed, smoking prevalence has remained consistently high in recent decades. Different national surveys consistently indicated that more than half of the Chinese population was exposed to second-hand smoke^30,34,35^. According to GBD results, smoking has been the major cause of cancer deaths from 1990 to 2017 in China (**Figure 4**). Cancer deaths attributable to smoking have increased by over 150%^9^. Given the time lag of several years from tobacco use and the development of cancer, a constant and very large smoking-attributable cancer burden in China is expected in the future decade. In addition, the smoking pattern among various age groups is cautiously optimistic. There is evidence that an earlier age of smoking initiation increases the risk of lung cancer^36,37^. Data from the National Health Services Surveys and Global Adults Tobacco Surveys in China show a steady rise in the smoking prevalence among adolescents^26,29,30^. This is corroborated with information from the China Health and Nutrition Surveys, which showed that the age of smoking initiation in the Chinese population decreased over time^38^. According to the Global Youth Tobacco Survey in China, 6.9% of youth (ages 13–15 years) were tobacco users (males: 11.2%; females: 2.2%) in 2014^39^. Over 80% of youth were not prevented from buying cigarettes, although laws have banned tobacco sales to those under 18 years of age in China^39^.

**Table 2:** Summary for smoking prevalence from nationally representative surveys

Nationally representative surveys of smoking in China	Year	Current smokers (%)		
		Overall	Male	Female
National Survey on Smoking^25^	1984	33.9	61.0	2.0
National Smoking Prevalence Survey^31^	1996	35.3	63.0	3.8
China Chronic Disease and Risk Factor Surveillance Survey^32,33^	2002	31.4	57.4	2.6
	2013–2014	27.3	51.8	2.3
China National Nutrition Survey^28^	2002	23.6	49.6	2.8
	2012	26.5	50.4	2.0
National Health Services Survey^29^	2003	26.0	48.9	3.2
	2008	25.1	48.0	2.6
	2013	25.7	49.3	3.1
Global Adults Tobacco Survey in China^26,30^	2010	28.1	52.9	2.4
	2015	27.7	52.1	2.7
	2018	26.6	50.5	2.1

Since the Chinese government signed the WHO Framework Convention on Tobacco Control (FCTC) in 2003, efforts have been made to control tobacco consumption, such as raising taxes and banning smoking in indoor public places^40^. The positive effects of these actions have begun to emerge, but are not enough^41,42^. For example, an increased tax on cigarettes is seen as the most effective tobacco control intervention^43^, but the tax in China still does not meet FCTC’s requirement. A modeling study estimated that smoking prevalence in China would reach 46.5% in males and 1.3% in females under the current policy. If China adopts all FCTC policies, the smoking rate would reach 28.2% in males^44^. The Chinese government aims to reduce smoking prevalence to 20% in the population aged 15 years or older by 2030. To achieve this goal, stronger policies relating to tobacco control are needed^45^.

#### Physical activity

Numerous epidemiological studies are conducted each year to examine whether physical activity reduces the risk of several types of cancers. The evidence so far is strong for breast, colon, and endometrial cancers and limited for lung, liver, and esophageal cancers^46^. It was estimated that 3.0% of colorectal cancer deaths in Chinese males were attributed to the lack of physical activity, in 2014. Among females, 4.3% of colorectal deaths, 8.8% of uterine cancer deaths, and 11.6% of breast cancer deaths were attributed to physical inactivity in China^19^. The number of cancer deaths attributable to low physical activity has increased by 1.5-fold (**Figure 4**).

The American Cancer Society recommends 150–300 min of moderate intensity physical activity or 75–150 min of vigorous intensity physical activity per week^47^. A series of national surveys by the China Physical Fitness Surveillance Center revealed that the proportion of Chinese adults meeting the minimum recommendation of physical activity for leisure increased from 17.2% in 2000 to 22.8% in 2014^48^. In contrast, data from the China Health and Nutrition Survey showed a worrying trend, showing that physical activity levels experienced a steady decrease^49–51^, which is expected due to mechanization, the transformation of economic structure, and changes in the living environment during the urbanization process. These surveys of physical activity rely on self-reports of activity, where accuracy is limited by high random error and systematic bias. High quality prevalence data are still urgently needed to evaluate the nationwide level of physical activity. In 2016, the Chinese government issued a new National Fitness Plan^52^ (2016–2020). According to the plan^52^, the per capita area of sports fields is expected to reach 1.8 square meters and the number of the people engaging in physical exercises at least once per week will increase to 700 million by 2020. The efficacy of this national plan needs to be evaluated in future studies.

#### Diet

Diet is an important risk factor for cancer, having a role in energy balance and in other biological mechanisms independent of body weight. However, determining a clear association of diet and nutrition with cancer is challenging, which could be attributed to the long latency between exposure and outcome, the complexity of dietary components, and the inevitable measurement errors. In collaboration with the American Institute for Cancer Research (AICR), the World Cancer Research Fund (WCRF) published the third expert report in 2018, which was widely recognized as the most authoritative and up-to-date resource for cancer prevention using diet and nutrition^46^. Independent expert panels convened by the WCRF/AICR evaluated the latest evidence from the Continuous Update Project and gave their recommendations.

The WCRF/AICR 2018 report concluded that greater dietary fiber and whole grain intake “probably” protect against colorectal cancer. Dietary fiber can influence bacterial species in the colon, whose fermentation products are considered to have anti-proliferative effects^53^. The proportions of colorectal cancer deaths attributable to low dietary fiber consumption were estimated to be more than 10% among both males and females in China^19^. Another important risk factor for colorectal cancer is a diet high in calcium and dairy products. The evidence that the intake of dairy products decreases colorectal cancer risk is considered “probable” by the WCRF/AICR. Estimates attribute 16.6% and 17.8% of colorectal cancer deaths to low dietary calcium consumption in males and females, respectively^19^. Red meat is an umbrella term referring to beef, veal, pork, lamb, goat, mutton, and horse meat, which are rich in heme iron. The most recent WCRF/AICR report considers the evidence “probable” that red meat consumption increases the risk of colorectal cancer. It was estimated that 4.4% of colorectal deaths in males and 3.3% in females could be attributed to red meat consumption in China^19^. Processed meat is considered “convincingly” associated with a higher risk of colorectal cancer. Similar to red meat, processed meat has high levels of fat and heme iron, which produces heterocyclic amines and polycyclic aromatic hydrocarbons during cooking at high temperatures. Almost 1% of colorectal cancer deaths in the Chinese population could be attributed to processed meat^19^.

Vegetables and fruits contain numerous potential anti-tumorigenic agents, including fiber, minerals, carotenoids, phenols, indoles, etc. In addition, intervention studies showed that consumption of vegetables and fruits could contribute to weight management^54^. They may also indirectly decrease cancer risk by influencing body weight. The WCRF/AICR report concluded that the intake of vegetables and fruits is “probably” associated with a lower risk in aerodigestive cancer^46^. Low fruit intake was ranked the top risk factor for cancer deaths in females and ranked as third in males. The WCRF/AICR report stated that salt-preserved food is “probably” related to the higher risk of stomach cancer^46^. It is probable that the formation of N-nitroso compounds and the change of gastric mucus viscosity are responsible for the increased risk of stomach cancer. The recent estimates in China attributed 3.6% of stomach cancer deaths in males and 3.7% in females to salt-preserved vegetable consumption^19^.

Driven by dramatic economic and social transformations, the dietary pattern among the Chinese population has shifted significantly^55^. Nationally representative data from the China National Nutrition Surveys (CNNS) conducted in 1982, 1992, 2002, and 2012^28,56^, and the 1989–2015 China Health and Nutrition Survey^57^ help delineate the nutrition transition in China. Data from the two large surveys consistently showed that the consumption of animal-source foods rapidly increased in China^28,58^. A steady increase in red and processed meat consumption was also observed^59^. Corresponding to this trend, the GBD study estimated that there was a 10-fold increase in cancer deaths attributable to red and processed meat from 1990 to 2017^9^. According to the CNNS, the intake of vegetables in the Chinese population decreased from 316.1 g/d in 1982 to 269.4 g/d in 2012^28^. In contrast, the consumption of fruits showed a gradual increase over the past decades^56,60^. In the context of a sharp increase in overall cancer deaths, the number of cancer deaths attributable to low fruit consumption remained steady. To relieve the huge burden of diet-related NCDs, the government released the National Nutrition Plan (2017-30) in 2017^61^. The plan emphasized salt reduction and weight management in Chinese residents, and stressed the role of nutritional intervention in the prevention and treatment of diet-related NCDs.

#### Alcohol consumption

Similar to tobacco, alcohol is carcinogenic, and abundantly consumed worldwide. According to the report by WCRF in 2018^46^, alcohol consumption increases the risk of at least 6 types of cancer, including esophagus, breast, colorectum, stomach, liver, and mouth, pharynx, and larynx cancer. Wine culture is well-established in China for thousands of years, which has a unique position in Chinese culture. Since the government abolished the monopoly management of alcohol in the 1980s, China has witnessed a striking increase in alcohol consumption^62^. A survey during 2004–2008 covering 0.5 million people reported that 33% of males and 2% of females drank alcohol at least weekly. Data from the 2007 China Chronic Disease and Risk Factor Surveillance showed that the mean consumption was 47.8 g per drinking day among male drinkers^63^. According to the study by Chen et al.^19^, alcohol consumption was the fourth top risk factor contributing to cancer deaths among Chinese males. Almost 20% of esophageal cancer deaths in males were caused by alcohol drinking in China^19^. According to the GBD study, alcohol consumption remained the third top risk factor contributing to cancer deaths during 1990 and 2017 (**Figure 4**). Reducing alcohol consumption is listed as one of the WHO Best Buys for controlling NCDs^64^. However, the alcohol control policy is poor in China, when compared to tobacco control. Alcohol drinking is obligatory during celebrations, festivals, and business meetings in China. Alcohol legislation is one potential way to manage alcohol production, marketing, and consumption^65^.

### Clinical risk factors

#### Excess body fat

Excess body fat occurs when surplus energy intake and low energy expenditure lead to positive energy balance. Being overweight or obese has been shown to affect diverse systematic functions, which could promote carcinogenesis, including chronic inflammation, inhibition of apoptosis, hormone metabolism, and oxidative stress^66^. In 2016, an expert panel report^67^ from the IARC showed that excess body fat increases the risk of cancer at several sites including the breast, endometrium, kidney, esophagus, colon, rectum, gastric cardia, liver, gallbladder, pancreas, and ovary. In addition, results from intervention studies showed that weight loss could have potential benefits for breast and endometrial cancer patients^68^.

Various anthropometric measurements of body fat include computed tomography, magnetic resonance imaging, body mass index (BMI), and waist circumference. The latter two are used widely in population surveys due to the low cost and ease of use. According to the Chinese guidelines for prevention and control of overweight and obesity^69^, overweight is defined as a BMI of 24.0–27.9 kg/m^2^, and obesity is defined as a BMI ≥28 kg/m^2^ in the Chinese population, based on a meta-analysis of associations of BMI with risk factors for cardiovascular disease among Chinese adults^70^, which differs from the World Health Organization definition of overweight (25.0–29.9 kg/m^2^) and obesity (BMI ≥30 kg/m^2^). The epidemic of obesity has become an increasingly important issue across the globe over the past decades. The prevalence of obesity worldwide has doubled from 1980 to 2008, with 4.8% increasing to 9.8% among males and 7.9% increasing to 13.8% among females^71^. In China, the increase in obesity rate has been observed regardless of the cut-off values used^48,72–74^. Data from the National Chronic Disease and Risk Factor Surveillance show that when referring to Chinese criteria, the overall prevalence of obesity in Chinese adults was 14.0% in 2014, which has tripled since 2004^75^. Geographic heterogeneity of obesity prevalence was evident at the provincial level, ranging from 6.4% in Guangxi to 24.9% in Beijing among females, and from 4.4% in Hainan to 26.6% in Beijing among males^75^. Such an increase in the prevalence of obesity is driving the rise of the burden of NCDs in China. The GBD study estimated that cancer deaths caused by excess body weight increased by more than 250% in China over the 3 decades^9^. Excess body weight has been ranked as the fourth leading risk factor causing cancer deaths in females. Approximately 16% of breast cancer deaths in females have been reported to be associated with excess weight and obesity^19^. It should be noted that the obesity epidemic has caused and will continue to cause a considerable cancer burden in China. The increase in the obesity rate over the past decades is the consequence of the abovementioned nutrition transitions and changes in physical activity in China. Thus, strategies to improve the dietary structure and physical activity levels should decrease the growing problem of excess body fat. In addition, evidence-based interventions for obese patients should be considered^76^.

#### Diabetes

Diabetes and cancer have many common risk factors, such as obesity, physical inactivity, and smoking. In addition, metformin use may affect the cancer incidence and mortality in patients with diabetes^77^. Thus, compared with the identification of other risk factors, it could be more difficult to determine whether a causal relationship exists between cancer and diabetes. In 2015, an umbrella review of meta-analyses concluded that there was robust evidence for the association of diabetes with breast, colorectal, liver, and endometrial cancers^78^. Possible mechanisms underlying diabetes and cancer are not clearly understood, but an increasing number of animal studies have proposed some pathways involved in inflammation, endoplasmic reticulum stress, and deregulation of autophagy^77,79^. In parallel with the increasing obesity rate in China, the prevalence of type 2 diabetes is rising dramatically. In 1980, less than 1% of Chinese adults were diagnosed with diabetes^80^. This figure rose to 2.5% in 1994^81^ and then increased to 5.5% in 2001^82^, which was followed by a further increase to 9.7% in 2007^83^ and 11.6% in 2010^84^. Over recent years, the prevalence of diabetes has remained at a high level, with the rate reaching 10.9% in 2013^85^ and 11.2% in 2017^86^. The rising trend of diabetes may be exaggerated to some extent due to the different sampling methods and diagnostic criteria used in national surveys. However, the rapid increase in diabetes is real in China. The key to diabetes management lies in sufficient glycemic control, which has been achieved by only one-third of diabetes patients^85^. Together with the increasing diabetes rate, the number of cancer deaths attributable to high fasting plasma glucose has tripled during 1990 and 2017^9^. Among women, 16.4% of uterine cancer deaths in 2014 could be attributed to diabetes. For liver cancer deaths in China, 10.9% in males and 8.8% in females could be avoided by preventing diabetes^19^. Curbing the epidemic of diabetes needs better diabetes prevention and management. The Da Qing Diabetes Prevention Outcome Study conducted among the Chinese population showed that lifestyle interventions significantly decreased the risk of diabetes in high risk individuals, which provides strong support for diabetes prevention^87^. Primary care is at the core of diabetes management, but is still underdeveloped in China. To solve that problem, some large hospitals with high quality medical resources and community clinics are integrated into the so-called “hospital group”^88^.

#### Infectious diseases

In 1911, Peyton Rous^89^ found that certain types of cancers in chickens were transmitted by a virus, which is now known as Rous sarcoma virus (RSV). Since that time, infections with some viruses, bacteria, and parasites have been identified as risk factors for certain types of human cancers. It is also worth noting that studies of RSV directly led to the discovery of oncogenes^90^. Just like RSV, some viruses such as hepatitis B virus (HBV)^91^ and human papillomavirus (HPV)^92^ directly induce genetic instability and cause uncontrolled cell growth. Infections with certain microbes could also suppress the immune system and cause long-term inflammation, which can eventually lead to cancer. Among those oncogenic microbes, HBV and HPV have received the most attention because they are widely prevalent and preventable by vaccines. According to a study by Chen et al.^19^, HBV infection contributed to 55.6% of liver cancer deaths among males, and 46.5% deaths among females in 2014. Almost all cases could be attributed to HPV infection, so cervical cancer is thus regarded as one of the most preventable types of cancer. In 2018, the WHO Director-General called for action towards the elimination of cervical cancer^93^. The vaccine became available in 2006, but it took 10 years for the HPV vaccine to be introduced to China ^94^. The use rate of the HPV vaccine is low in China due to lack of public awareness and the high price^95,96^, while more than half of the target population has been vaccinated in some developed countries^97^. A modeling study estimated that China could achieve the ambitious goal of eliminating cervical cancer by the late 2050s, by improving financial support of HPV vaccination and cervical cancer screening^98^. In 2019, the first domestic HPV vaccine was approved by the Chinese government, which is expected to boost rate of HPV vaccination in China. The use of HBV vaccination is more prevalent. Since a trial involved in infant HBV vaccination was conducted in Qidong in 1983^99^, the use of HBV vaccination was progressively expanded in China^100^. HBV vaccination has now been integrated into routine infant immunization in China. As a powerful weapon against liver cancer, the increased use of HBV vaccinations has contributed to the decrease of liver cancer burden in China^14^. For example, the HBV vaccination in Shanghai has decreased the numbers of HBV cases by 85% and halved the hepatocellular carcinoma incidence^101^.

### Environmental risk factors and occupational exposure

#### Air pollution

Increasing knowledge about the carcinogenic effects of air pollution has become available. Complex components of particulate matter exhibit high carcinogenic potential through several mechanisms^102^. In 2013, outdoor air pollution and particulate matter from outdoor air pollution were listed as carcinogenic to humans by the IARC Working Group^103^. A heavy haze is common in northern China, which has caused great public concern. Coinciding with the largest economic growth, China also became one of the countries suffering from the worst air pollution^104^. A report from the National Cancer Center attributed 14.4% of lung cancer deaths to PM_2·5_ air pollution in China. The GBD study estimated that the number of cancer deaths related to ambient particulate matter pollution increased by more than 300% from 1990 to 2017 (**Figure 4**). To decrease serious air pollution, legislation was passed by the government. In 2013, the National Action Plan on Air Pollution Prevention and Control (2013–2017) was announced, which was thought to be the most influential environmental policy in China over the last decade. The Action Plan defines 10 strict measures, including controlling the production capacity of high pollution industries and promoting public transport development.

The remarkable success of the Action Plan has been documented by air quality monitoring sites. Monitoring data from 74 key cities in China showed that annual average concentrations of PM_2·5_ decreased by one-third from 2013 to 2017^105^. Air quality improvement has benefits beyond those for lung cancer control, and recently a study from China indicated that decreasing PM_2.5_ air pollution since 2013 has been associated with increased life expectancy^106^. It was estimated that, compared with 2013, more than 47,000 deaths were avoided in 2017 due to improvements in air quality in 74 key cities of China^105^. However, reaching the WHO-recommended annual average PM_2.5_ concentration of 10 μg/m³ in China requires a long-term effort. In 2018, the Chinese government released the Three-year Action Plan for Winning the Blue Sky War (2018–20), the second phase of the original Action Plan. With the challenge of maintaining economic growth, China’s attempt to reach the target is thought to be challenging.

In addition to the outdoor air pollution, a growing body of evidence has identified household air pollution as a risk factor for lung cancer. The IARC also classified household coal combustion as carcinogenic to humans^107^. Xuanwei, a county in the Yunnan province of China, became a focus in the 1980s due to its high lung cancer mortality. Indoor air pollution by the domestic use of smoky coal was established as a principal contributor to lung cancer deaths in Xuanwei^108,109^. Household energy sources are determined by social and economic factors^110^. With the rapid urbanization and the rise of income, the mean household air pollution exposure has decreased in China^111^. Likewise, the number of cancer deaths attributable to household air pollution from solid fuels has decreased by 32.1% between 1997 and 2017 (**Figure 4**). However, many residents in rural areas still use solid fuel for cooking and heating, and are exposed to high household air pollution in China^112^. In 2017, the government released the Clean Heating Plan for Northern China in Winter (2017–21) to substitute residential solid fuels with electricity or pipeline-based natural gas in the Beijing-Tianjin-Hebei area. The initial benefits of this policy are already being seen by rural residents, especially by females, who tend to do the cooking^113^.

#### Occupational exposure

In the 18th century, Percivall Pott, a physician from England, found that occupational exposure to soot was the cause of scrotal cancer (later called testicular cancer) in young chimney sweeps^3^. His finding is recognized as a milestone in occupational medicine. A century and a half later, the carcinogen in soot was identified as polycyclic aromatic hydrocarbons. In recent decades, the number of identified occupational carcinogens has increased^114^. According to a GBD study, asbestos was the top occupational hazard causing cancer deaths in 2017, replacing silica (**Figure 4**). China ranks second in the consumption of chrysotile asbestos worldwide^115^. An increasing trend of malignant mesothelioma, the main cancer associated with asbestos exposure, was observed in China during 2000 and 2013^116^. Although the government required protective measures for workers exposed to chrysotile in 2014^117^, stricter rules on the use of chrysotile asbestos is expected by the healthcare industry^118^. In 2001, the Law on the Prevention and Control of Occupational Diseases was issued. One year later, the Work Safety Law, another important law involved in controlling occupational hazards, became effective^119^. However, occupational health in the workplace is still an important public health issue, which deserves more attention. Occupational health was listed as one of the major health campaigns in the action plan of Healthy China 2030, denoting efforts of the Chinese government to protect the health of workers.

## Strategies for cancer prevention

**Figure 5:**
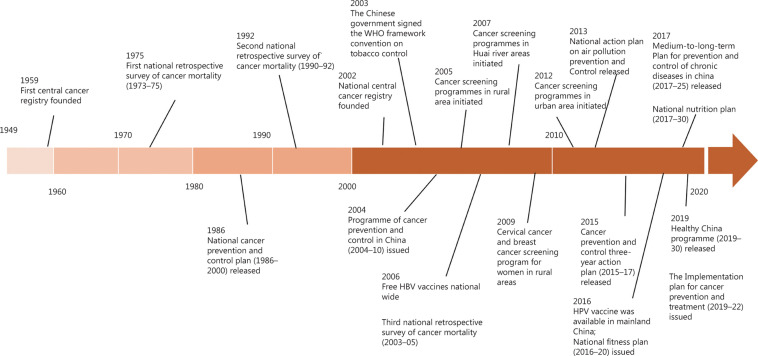
Timeline of key policies in cancer prevention and cancer-related risk factor control in China.

Without support and planning by the government at the national level, curbing the cancer epidemic will be impossible for such a large and populous country like China. Since the establishment of the People’s Republic of China in 1949, an increasing number of policies, programs, and plans have been issued by the Chinese central government to prevent and control cancer and cancer-related risk factors nationwide (**Figure 5**). Tracing back to the 1950s, the high incidence of esophageal cancer in Linzhou raised the concern of the central government, where the first Chinese cancer registry was established in 1959^5^. During the following decades, preventive interventions, esophageal cancer screening, and advanced treatment were introduced in Linzhou. The first national retrospective survey of cancer mortality (1973–1975) was then conducted in China in the 1970s. This survey identified several high risk areas like Linzhou, where the cancer mortality rate was far higher than the national average. Priority was allocated to these high risk areas, where cancer control activities were established, including primary prevention, cancer screening, and scientific research. Valuable population-based evidence was generated from these high cancer areas^120,121^. Significant progress has been made, but some challenges remain. In 2016, improving health was elevated to a new height in China. The Healthy China 2030 blueprint was unveiled at a national health conference. Three years later, an ambitious action plan was released, which specified tasks of 15 major health projects, including prevention and control of cancer. Recently an Implementation Plan (2019–2022) targeting cancer prevention and treatment under Healthy China 2030 has emphasized the need for control of cancer-related risk factors and early diagnosis of cancer.

### Primary prevention

Compared with cancer screening and treatment, primary prevention should be the most cost-effective way to curb the growth in cancer burden. Other NCDs, including cardiovascular diseases and diabetes, share many overlapping risk factors with cancer such as smoking, obesity, physical inactivity, and poor nutrition. A prospective cohort study showed that myocardial infarction survivors who develop heart failure have an increased risk of cancer compared with those patients without subsequent heart failure^122^. Excess benefit for other NCDs could be expected when primary prevention works. Additionally, public health interventions always have a positive effect that extends beyond the target population and influences the population, who did not directly receive the intervention. A typical example of the spillover effect is herd immunity provided by vaccines. A USA study suggested that the increased use of the HPV vaccine could provide unvaccinated men with herd immunity against HPV infection^123^.

Behavioral risk factors are the main contributors to cancer in China. However, there are many challenges in changing individual health behaviors. Some theories of behavioral changes stress the importance of knowledge and awareness^124^. The action plan of Health China 2030 sets a goal of increasing the population with health literacy from 14.18% to 22% in 2022 and 30% in 2030. No nationwide representative surveys described the level of health literacy related to cancer prevention in Chinese residents. Several regional surveys suggested poor knowledge about cancer-related risk factors and cancer screening in the Chinese population^125–127^. Media campaigns aimed at smoking and sun protection have been successful in increasing knowledge, changing social norms, and changing individual behaviors in other countries^128–130^. To raise public awareness of cancer control, the China Anti-Cancer Association initiated the “4·15 National Cancer Week” campaign in 1995, involving various activities undertaken every year^131^. With the rapid growth of the mobile internet, social media, podcasts, and personal blogs are important channels of dissemination of evidence-based cancer prevention information.

However, the relationship between health knowledge, attitude, and behavior is not linear. The classical example is the high prevalence of smoking among doctors and medical students^132^. Educational interventions targeting individual behavioral changes for primary prevention have not been effective in preventing the cancer epidemic. The risk factor distribution in the population and the effectiveness of primary prevention are influenced by many social determinants. Diet, for example, is closely associated with the local food environment^133^. In addition, people of high socioeconomic status (SES) are likely to consume nutrient-dense food such as lean meats, fish, fresh fruits, and vegetables^134^. Thus, primary prevention is not practical without preventive strategies and policies used across multiple sectors of the entire society. Public health in the modern era involves cross-sectional cooperation and the improvement of social determinants of health, to change policies, systems, and the environment^135^. There is a growing body of evidence showing the effectiveness of environmental interventions on eliminating risk factors. A study from Mexico indicated a reduction of soft drink consumption in a cohort after the implementation of a sugar-sweetened beverage tax^136^. A study from China showed that higher cigarette prices led to a decline in smoking initiation^137^. Furthermore, evidence has shown that downstream interventions tend to increase health disparities more than upstream interventions^138^. For example, media campaigns to promote smoking cessation tend to be less effective for disadvantaged populations. Compared with the groups of higher socioeconomic status (SES), people of lower SES are less likely to seek subsequent treatment, even though they receive the same media message^139^.

In 2016, the “prevention first” principle was once again stressed at the highest level national health conference of China. In addition, different from previous working principles of health development, “health in all policies” was included as the working principle in the new era. This provided a solid political foundation for integrating health considerations into policymaking across government sectors in China. More upstream interventions for the primary prevention of cancer could be expected from the Chinese government.

### Secondary prevention

In addition to screening for breast, cervical, and colorectal cancers, which is thought to be cost-effective and recommended in many countries^140^, China also implements screening programs for cancers with high burdens, including stomach, esophageal, lung, liver, and nasopharyngeal cancers. Organized screening programs were limited in several high risk areas in the last century, which benefited several generations. For example, 54 cervical cancer and 153 cases of cervical intraepithelial neoplasia were detected in the cervical cancer screening program implemented in Jing’an county, Jiangxi province from 1991 to 2004^141^. At the time of writing, many organized cancer screening programs covering thousands of people have been implemented in China (**Figure 5**). Risk-based screening strategies are used in most programs to increase cost-effectiveness. Current screening programs are still not sufficient to alleviate the cancer burden in China, although the positive effect of those screening programs has shown^142,143^. The Implementation Plan (2019–22) of Cancer Prevention and Treatment calls for guidelines of early detection to standardize screening for upper gastrointestinal, cervical, and colorectal cancers. The plan also addresses the expansion of cost-effective cancer screening tailored to local profiles and population risks. It is anticipated that more of the Chinese population will benefit from cancer screening.

## Conclusion and perspective

Changes in modifiable risk factors for cancer, especially diet, obesity, diabetes, and air pollution, have contributed to the cancer transition in China. A heavy burden of upper gastrointestinal cancer and a rising burden of colorectal, prostate, and breast cancers exist together during the transition stage. Lung cancer became the top cause of cancer-related deaths, coupled with tobacco use as the most important contributor to cancer deaths. Tobacco control should be implemented first to decrease both active and passive smoking. Despite the gradual decrease in smoking and improvements of air quality, a huge lung cancer burden in China is predicted in the next decade, considering the time lag of several years from exposure to the development of cancer. It is encouraging that the burden of esophageal, stomach, and liver cancers has dropped significantly. Credit goes to the elimination of risk factors, such as the change of dietary habits and the spread of HBV vaccination. The continuous decrease in the burden of upper gastrointestinal cancer could be expected in the following decades^144,145^. Recent healthcare policies under Healthy China 2030 also have tried to promote standard cancer screenings and treatments. The measurable reduction of cancer burden in China will require joint efforts from governments, public health organizations, and individuals to reduce modifiable risk factors, promote population-wide screening, and improve cancer treatment outcomes.
